# Supplementary material for: The causal role of circulating inflammatory markers in osteoporosis: a bidirectional Mendelian randomized study
Source: Front Immunol. 2024 Jul 18;15:1412298. doi: 10.3389/fimmu.2024.1412298 (PMC11291241; doi:10.3389/fimmu.2024.1412298)
Supplement: Supplementary file 1 [file DataSheet_1.zip › supplementary material/OSTEOPOROSIS-CIM/GCST90274817.tsv.gz_leave_one_out.pdf]

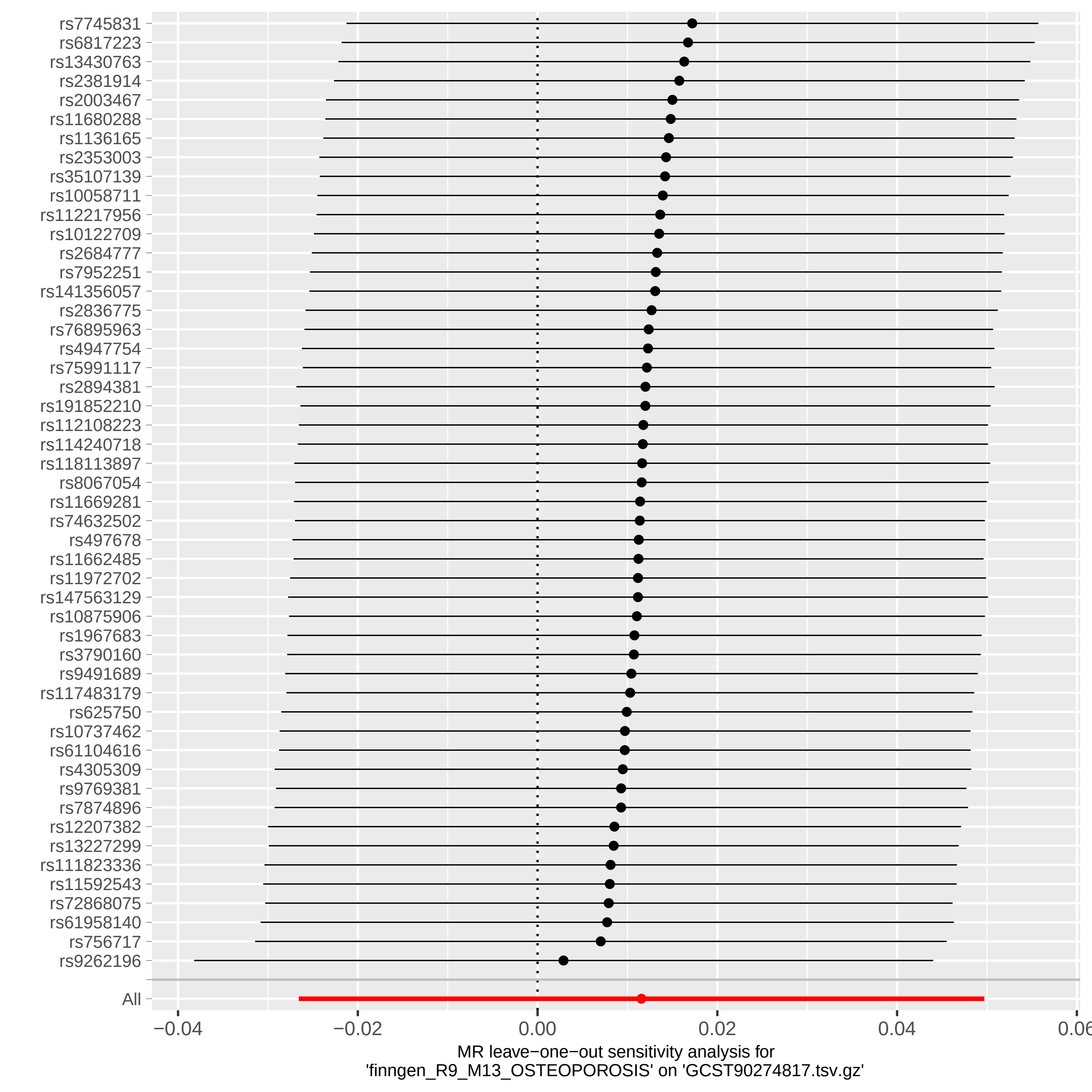
rs7745831
rs6817223
rs13430763
rs2381914
rs2003467
rs11680288
rs1136165
rs2353003
rs35107139
rs10058711
rs112217956
rs10122709
rs2684777
rs7952251
rs141356057
rs2836775
rs76895963
rs4947754
rs75991117
rs2894381
rs191852210
rs112108223
rs114240718
rs118113897
rs8067054
rs11669281
rs74632502
rs497678
rs11662485
rs11972702
rs147563129
rs10875906
rs1967683
rs3790160
rs9491689
rs117483179
rs625750
rs10737462
rs61104616
rs4305309
rs9769381
rs7874896
rs12207382
rs13227299
rs111823336
rs11592543
rs72868075
rs61958140
rs756717
rs9262196
All
−0.04
−0.02
0.00
0.02
0.04
0.06
MR leave−one−out sensitivity analysis for
'finngen_R9_M13_OSTEOPOROSIS' on 'GCST90274817.tsv.gz'
